# Data-driven testing program improves detection of COVID-19 cases and reduces community transmission

**DOI:** 10.1038/s41746-022-00562-4

**Published:** 2022-02-11

**Authors:** Steven J. Krieg, Carolina Avendano, Evan Grantham-Brown, Aaron Lilienfeld Asbun, Jennifer J. Schnur, Marie Lynn Miranda, Nitesh V. Chawla

**Affiliations:** 1 Lucy Family Institute for Data and Society, University of Notre Dame, Notre Dame, IN 46556 USA; 2 Children’s Environmental Health Initiative, University of Notre Dame, Notre Dame, IN 46556 USA

**Keywords:** Computer science, Epidemiology

## Abstract

COVID-19 remains a global threat in the face of emerging SARS-CoV-2 variants and gaps in vaccine administration and availability. In this study, we analyze a data-driven COVID-19 testing program implemented at a mid-sized university, which utilized two simple, diverse, and easily interpretable machine learning models to predict which students were at elevated risk and should be tested. The program produced a positivity rate of 0.53% (95% CI 0.34–0.77%) from 20,862 tests, with 1.49% (95% CI 1.15–1.89%) of students testing positive within five days of the initial test—a significant increase from the general surveillance baseline, which produced a positivity rate of 0.37% (95% CI 0.28–0.47%) with 0.67% (95% CI 0.55–0.81%) testing positive within five days. Close contacts who were predicted by the data-driven models were tested much more quickly on average (0.94 days from reported exposure; 95% CI 0.78–1.11) than those who were manually contact traced (1.92 days; 95% CI 1.81–2.02). We further discuss how other universities, business, and organizations could adopt similar strategies to help quickly identify positive cases and reduce community transmission.

While schools, businesses, and other intuitions seek to continue normal operations, COVID-19 remains a global threat—especially as global vaccine rollouts remain in progress and the ongoing emergence of SARS-CoV-2 variants introduces new uncertainties^1–3^. These organizations must therefore be prepared to detect and mitigate its risk to their people and activities. In this report, we share key lessons learned from an adaptive COVID-19 testing program implemented at the University of Notre Dame. The adaptive testing program utilized two different, data-driven network models to quickly and accurately predict which students had an elevated risk of contracting COVID-19 and should be called proactively for testing. Both models utilized a social network representation of the university community in which each node represented a person (our analysis focuses exclusively on students) and each edge represented a connection between two people (e.g., roommates, enrolled in the same course, active on the same sports team). The first model predicted individual student risk directly, and the second model predicted which pairs of students were most likely to be close contacts. The key difference between the two lies in the problem formulation: the first model was trained for a node-level task (classifying students as high-risk or low-risk using prior COVID-19 test results as training data), while the second was trained for an edge-level task (predicting contact tracing relationships between students using contact tracing records from the previous semester as training data). While both models operated within the same social network, the difference in model inputs and optimization strategy resulted in models that were diverse and complementary, able to identify high-risk individuals within the campus network while reducing the overhead of manual contact tracing. The success of this program suggests that machine learning strategies can improve the effectiveness of surveillance testing or other efforts to efficiently distribute testing resources and reduce community transmission. Importantly, both the node classification and link prediction models produced useful predictions. When we also consider the flexibility of social network representations, these results suggest that even in the absence of data on positive tests organizations could make use of other available data to model transmission risk via activity in a social network. In our university context, this data included shared classes, sports teams, and dormitories. In workplaces, enterprise social network (ESN) analysis has used data such as shared meetings, formal organizational structure, project assignments, office proximities, and virtual interactions via e-mail or instant messaging systems to great effect on other tasks^4–6^. Given the present uncertainties about the COVID-19 pandemic, making full use of available data and machine learning techniques may be more important than ever in mitigating future outbreaks.

The adaptive testing program was one of many COVID-19 mitigation strategies implemented throughout the 2020–21 academic year at the University of Notre Dame^7^. During the fall of 2020, 1,556 students (12.0%) and 200 faculty and staff tested positive from a total of 88,283 tests. In just the first four weeks of the spring 2021 semester (Feb. 3 through Mar. 2, 2021), another 734 students (5.7%) and 34 faculty and staff tested positive from a total of 57,661 tests. This provided a rich set of test results, contact tracing interviews, and symptom reporting to use as training data. The situation also necessitated urgent intervention—especially with respect to asymptomatic and presymptomatic cases, which contributed significantly to community transmission^8^. Thus a targeted and data-driven adaptive testing program was initiated on March 3, 2021 to *supplement* general surveillance testing, manual contact tracing, quarantine/isolation protocols, and self-reported health checks with more targeted and data-driven testing.

## Results

### Positive tests

The adaptive testing program began on March 3 and finished on April 30. Cohorts were tested daily with only a few exceptions (e.g., no adaptive tests were administered from April 17 to 19 to provide testing staff with time off during Easter weekend). During this period 115,224 total tests were administered to students: 79,932 (69.4%) to the general surveillance cohort, 20,862 (18.1%) to the adaptive cohort, and the remaining 14,430 (12.5%) to other cases such as students who reported symptoms or were contact traced. Of the 12,211 active students at the university, 11,833 were tested at least once via general surveillance and 6,459 via adaptive testing. A total of 641 students tested positive: 297 (46.3%) during a general surveillance appointment, 111 (17.3%) during an adaptive testing appointment, and the remaining 235 (36.3%) during symptomatic appointments. As summarized in Table 1, the general surveillance cohort thus produced a positivity rate of 0.37% (95% CI 0.28–0.47%), while the adaptive cohort produced a positivity rate of 0.53% (95% CI 0.34–0.77%).

**Table 1:** Summary of testing results. NR and LP represent the students in the adaptive cohort who were predicted by either the node risk or link prediction model, respectively, and NR + LP represents the students who were predicted by both models.

Cohort	# Tests administered	Positive tests	Positivity rate
General surveillance	79,932	297	0.37% [0.28%, 0.47%]
Adaptive	20,862	111	0.53% [0.33%, 0.76%]
Adaptive (NR model only)	10,251	50	0.49% [0.42%, 0.56%]
Adaptive (LP model only)	8,089	32	0.40% [0.30%, 0.47%]
Adaptive (both NR + LP models)	2,608	21	0.81% [0.51%, 1.24%]

**Fig. 1:**
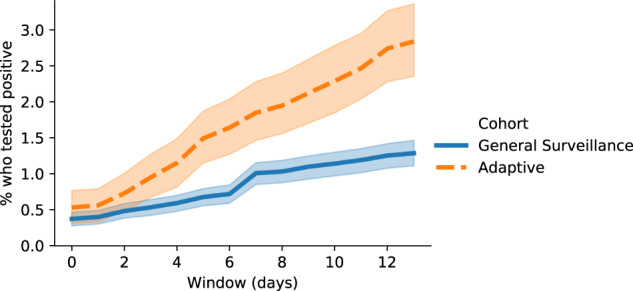
The percentage of students who tested positive during the initial appointment or a follow-up test within 14 days. Day 0 is the day they were selected for the cohort. Shaded regions indicate 95% confidence intervals.

Many students returned for a follow-up test within several days of being selected for the adaptive cohort. When we look beyond the same-day test results, students selected for adaptive cohorts were even more likely to test positive. Within five days of being called for testing, 0.67% (95% CI 0.55–0.81%) of the general surveillance and 1.49% (95% CI 1.15–1.89%) of the adaptive cohort tested positive—a 122% increase for the adaptive cohort. As Fig. 1 demonstrates, this gap in positivity rate between the two cohorts widens with the length of the follow-up window for at least 14 days after selection to the adaptive cohort.

### Differences between predictive models

**Fig. 2:**
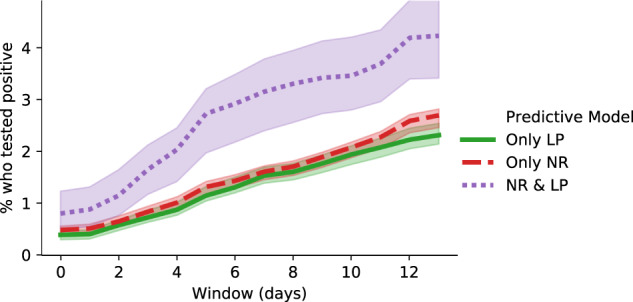
The percentage of students from the adaptive cohort who tested positive as predicted by the node risk (NR) and/or link prediction (LP) models within 14 days. Day 0 is the day they were selected for the adaptive cohort. Shaded regions indicate 95% confidence intervals.

We emphasize in Table 1 that the adaptive cohort is essentially comprised of three groups of students: 10,251 who were selected by only the node risk (NR) model, 8,089 who were selected only by the link prediction (LP) model, and 2,608 who were selected by both models. As shown in Fig. 2, students selected by both models were by far the most likely to test positive, with a positivity rate of 0.81% (95% CI 0.51–1.24%) on the initial test and 2.72% (95% CI 1.97–3.21%) testing positive within five days. They also tended to have the highest predicted risk from each model individually via connections like living on the same dorm floor or being in multiple courses with a student who had tested positive. Students selected by only one of the NR or LP models were at lower risk: positivity rates were 0.49% (95% CI 0.42–0.56%) and 0.40% (95% CI 0.30–0.47%), respectively, on the initial test; and 1.32% (95% CI 1.17–1.42%) and 1.15% (95% CI 1.04–1.32%), respectively, tested positive within five days. We did not observe significant differences between the models with respect to the types of connections that produced high-risk predictions. For example, the conditional probabilities learned by the link prediction model had similar values to the edge weights learned by the node risk model (Table 3).

### Test timing and response rates

**Fig. 3:**
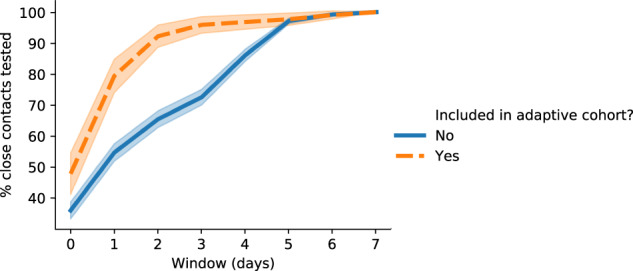
The distribution of average time to receive a test for students who were exposed to COVID-19 via close contact with another student. Day 0 is the day the exposing student tested positive. Shaded regions indicate 95% confidence intervals.

Another key finding was that the adaptive testing program resulted in a significantly shorter average time to test for close contacts. Of 1,907 contacts that were traced to the 641 positive cases, 1,483 were administered a test on campus within seven days. 188 were administered a test via selection to the adaptive cohort within an average of 0.94 days (95% CI 0.78–1.11), while the remaining 1,295—who were tested only after being notified of their exposure by contact tracers or the student who exposed them—were administered a test within an average of 1.92 days (95% CI 1.81–2.02). Figure 3 shows the full distribution of test timings for confirmed contacts.

We additionally found that the average same-day response rates were 78.1 and 95.0% for the general surveillance and adaptive cohorts, respectively. The differences between undergraduate, graduate, and professional students were marginal (79.4, 78.1, and 82.1%, respectively) for the general surveillance cohort. For the adaptive cohort, the differences between means were more significant (96.8, 85.6, and 92.7%, respectively); however, the number of adaptive tests administered to graduate and professional students was only 442 (2.3%) and 631 (3.3%), respectively. While the general surveillance cohort was least likely to respond on Saturdays and Sundays (61.2%), only 8.3% of these appointments were scheduled on these days, and the response rate for weekday appointments was only 79.6%. Thus in all cases, the response rate for adaptive testing was higher than for general surveillance.

## Discussion

Rapidly identifying COVID-19 cases is of paramount importance to reducing community transmission^9^. While the adaptive program did produce higher same-day positivity rates, the model predictions gained value over time (Fig. 1). We suggest that this is explained largely by the following. First, the incubation period of SARS-CoV-2 means that students who are tested immediately (the next day) after a close contact tests positive may not yet have a detectable viral load^10^. Second, networks excel at modeling transmission dynamics in local communities (e.g., a dormitory floor). High-risk individuals should therefore be tested as soon as possible, but a single negative test does not allay the risk of further transmission through a third party. An ideal follow-up protocol should include cadenced re-testing for at least 14 days.

Prior to March 3, students were informed of the adaptive testing program and its data-driven approach to identifying individuals who were at high-risk. Therefore, some of the differences in response rates could be due to students’ perceiving an adaptive testing call as more important than a general surveillance one. However, it is also possible that adaptive testing appointments were correlated with individuals’ knowledge of potential exposure. It seems likely that both of these factors contributed to the increased response rate.

Because we can directly observe both the social network structure and the learned parameters for both models, their outputs are easily interpretable. For example, Tables 2 and 3 report the base risk scores for each student and the weights that govern risk flow to other students, as utilized by the node risk model in Eq. 1. After these parameters have been learned by the model via the evolutionary strategy, their contribution to individual predictions can be easily observed. Similarly, the conditional probabilities for the link prediction model (*σ*(*t*) in Eq. 3) could be easily observed once they had been learned on the training data. The university’s COVID-19 response team regularly utilized this interpretability to validate daily predictions, adjust the size of the testing cohort, and direct resources to the high-risk parts of the student body. In the context of our operational needs, this provided a substantial advantage over state-of-the-art models like graph neural networks^11^.

**Table 2:** Node base risk scores learned by the node risk prediction model. Higher scores indicate a greater transmission risk. To predict high-risk students, each student’s base risk score (*P*_0_) is propagated to neighboring nodes via Eq. 1. Q/I represents quarantine/isolation.

# Days prior	Positive test	Assigned to Q/I	Reported symptoms/exposure
1	0.9839	0.0294	0.0010
2	0.9208	0.1799	0.0283
3	0.9904	0.3884	0.0118
4	0.2026	0.2518	0.0010
5	0.0010	0.0043	0.0010
6	0.0679	0.1760	0.0010
7	0.3180	0.0518	0.0010

**Table 3:** Edge type weights learned by the node risk prediction model. These weights (*ω*_*t*,*i*_) are utilized in Eq. 1. Each value represents the risk for a student given that they share an edge of type *t* with another student at *i* hops in the network.

Edge type (*t*)	*ω*_*t*,1_	*ω*_*t*,2_
Shared address (roommates)	0.9978	0.9180
Shared dorm suite	0.3898	0.3718
Shared dorm floor	0.3064	0.2672
Shared dorm building	0.0868	0.0351
Enrolled in same course	0.0010	0.0010
Active on same sports team	0.8903	0.8566
Close contact	0.9806	0.0543

The shortcomings of contact tracing have been well-documented, including that the process requires a high amount of manual effort^12^ and that individuals can be reluctant to disclose their social activity^13^. The ability of an adaptive testing program to identify high-risk individuals and produce shorter times to test for close contacts can mitigate both of these problems. However, while our models are an effective supplement to manual contact tracing, they are not a replacement for it. There are many close contacts that were not predicted by our models, and many student behaviors that are not captured in a social network. Additionally, while we attempted to model the spread of symptoms in the social network (e.g., how likely is student A to test positive given that one of her classmates, student B, reported a fever), we found that symptom profiles were too noisy^8^ and did not improve predictive performance.

In addition to the imperfections of contact tracing, limitations of this work include that it only studied a relatively homogenous population of university students, a majority of whom are white (65%) and 19–22 years old (67%). Our analysis also assumes a closed community and cannot account for inter-community transmission, which can introduce noise into the predictive models. Further, social networks are limited in their ability to represent the full complexity of social activity in any community. For example, about 83% of reported close contacts shared one of the relationships listed in Table 3, meaning that the other 17% of close contacts could not be described by these data types. Finally, all machine learning is vulnerable to bias in the training data. For example, the lower node risk weights on days 5 and 6 in Table 2 may be attributable to our cadence for testing exposed students (follow-up tests on days 4 and 7 after exposure) rather than the true distribution of SARS-CoV-2 incubation^14^.

The simplicity of this adaptive testing approach lends itself well to generalization to other contexts. Universities and schools can easily construct a social network representation using data on class schedules, housing arrangements, and extracurricular activities. Workplaces and other organizations can draw on the rich history of ESN analysis to construct a social network that incorporates both formal (e.g., organizational reporting structures, meeting schedules, project assignments, physical work locations) and informal elements (e.g., e-mail, SMS, and instant messaging interactions)^4–6^. In an ideal scenario where both testing data and social activity is available, we suggest that training multiple models for different tasks will produce the most effective results. Specifically, COVID-19 testing produces node-level data and therefore supports node-level tasks; likewise, social activity produces edge-level data and thus supports edge-level tasks. As our results demonstrate, training models for both tasks can make predictions that are diverse and complementary. However, even in the absence of data on positive tests, our results show that a model optimized to predict close contacts serves as an effective proxy for predicting viral transmission directly. Such approaches may prove to be not only effective, but also necessary in order to rapidly detect positive cases and drive down community transmission.

## Methods

### Adaptive testing overview

During each day from March 3 and finished on April 30, 2021, two cohorts of students were sampled for surveillance testing. The first, which we call the general surveillance cohort, was determined by the students selecting a day of the week to be tested to ensure each student was tested once every week. The second, which we call the adaptive cohort, was selected via risk scores as predicted by two distinct machine learning models. Depending on the available testing resources (which increased substantially from fall to spring), at the beginning of each day the adaptive testing team chose the *n* students with the highest predicted risk from each model. Students who were already being tested for another reason such as reporting symptoms, living at the same address as a new positive case, having been contact traced, or having been tested twice already during the week (defined as a 7-day period from Monday to Sunday) were excluded from the adaptive sampling. Students in both the general surveillance and adaptive cohorts were notified of their selection for testing via email and text and administered either a saliva or nasal reverse transcription-polymerase chain reaction (RT-PCR) test at the university testing center (performed by a local commercial laboratory primarily using a Roche platform^15^) within 24 h of notification. When a test yielded a positive result, the student was instructed to isolate for 14 days. A response team, consisting of clinical and non-clinical personnel, conducted a brief phone interview to identify anyone who had been in close contact with the student. Confirmed close contacts were informed of exposure through contact tracers (without revealing the identity of the index case), instructed to quarantine, and administered a Sofia SARS Antigen Fluorescent Immunoassay (Quidel) rapid antigen test. If the antigen test produced a negative result, they were also administered a PCR test, instructed to quarantine, and called for additional tests on days four and seven after exposure. If all tests were negative by day seven, exposed students were released from quarantine. Students who were tested via the contact tracing procedure are excluded from both the general surveillance and adaptive cohorts.

### Network models

Foundational to the adaptive testing program was the modeling of the university as a heterogeneous network, a widely-used formalism in graph theory and network analysis^16^. Formally, we define a network *G* = (*V*, *E*), where *V* is a set of *n* nodes and *E* is a set of *m* edges. Each node *u* ∈ *V* represents one student, and each edge *e* ∈ *E* is a tuple (*u*, *v*, *t*) that represents a relation between two nodes *u* and *v* of type *t*. Possible relation types included two nodes sharing the same home or dorm address, being enrolled in the same course, playing the same team sport, sharing a dorm floor or building, and being confirmed as close contacts by a contact tracer. We additionally consider a weight function $w:E\to {\mathbb{R}}$ that maps each edge to a real-valued weight, or 0 if the edge does not exist. In our context, all edges have a weight of 1 except for students who were enrolled in the same course(s), in which case the edge weight was the number of courses they shared. This representation is thus a flexible and expressive means of modeling interactions among students in the context of a large community, and served as the input to the predictive models.

In designing the predictive models we prioritized the following principles:

1. Simplicity. The operational needs of the program were urgent, so we designed models that were relatively simple to develop, test, and deploy. This is true of both the learning algorithms and the underlying data.
2. Diversity. The combination of diverse models is the cornerstone of the success of ensemble methods in machine learning^17^. In our case, we encouraged diversity by optimizing each model for a different task within the campus social network: one model for a node-level task, and the other for an edge-level task. Taken together, the strengths and limitations of both models proved to be complementary in solving the operational problem.
3. Interpretability. The outputs of the predictive models were monitored by the adaptive testing team and subject to further operational constraints. For example, roommates of students who tested positive were already being called for testing, and so were excluded from adaptive testing selection. However, roommate connections contribute significantly to the campus social network structure. By designing models with interpretable outputs, we simultaneously enabled the models to make full use of the information provided by roommate connections and the adaptive testing team to make informed decisions with the aid of model outputs.

### Node risk prediction

The first model predicted risk at the node level by first assigning each node *u* ∈ *V* a base risk score *P*_0_ based on whether the student had recently tested positive, been assigned to quarantine/isolation, or reported COVID-19 symptoms (Table 2). Next, each node sent a portion of its risk to its neighbors based on the type of relationship they share. This risk propagation approach, known more generally as message passing, is foundational to many network inference tasks^18^. For a given node *u* we iteratively computed its final risk *P*_*i*_(*u*) according to the following:

1 $${P}_{i}(u)={P}_{(i-1)}(u)+\mathop{\sum}\limits_{t\in {{{\mathcal{T}}}}}\mathop{\sum}\limits_{v\in {{{{\mathcal{N}}}}}_{t}(u)}{\omega }_{t,i}{P}_{(i-1)}(v),$$

where *i* is a parameter defining the number of message passing iterations, ${{{\mathcal{T}}}}$ is the set of edge types, ${{{{\mathcal{N}}}}}_{t}(u)$ is the set of *u*’s neighbors via edge type *t* (i.e., (*u*, *v*, *t*) ∈ *E* for all $v\in {{{{\mathcal{N}}}}}_{t}(u)$), and *ω*_*t*,*i*_ is a learned weight parameter associated with edge type *t* at hop *i*. Intuitively, this means that during each iteration each node’s risk is updated with the weighted sum of the risk of its neighbors, where the weights are fixed for each combination of edge type and hop number. We treat *P*_2_ as the final score, such that each student’s risk is influenced by nodes up to two hops away in the network.

To learn the set of base risk scores *P*_0_ and weights *ω*_*t*,*i*_ (Tables 2 and 3), we utilized the following evolutionary strategy:

1. Given an initialized set of weights, target a random previous day in the semester, denoted as *d*.
2. Create a copy of the weights and make several small and random adjustments to them.
3. Simulate the testing results for day *d* via Eq. 1, and evaluate the predictions for both sets of weights.
4. Keep the weight set that more accurately predicted which nodes tested positive on day *d*.
5. Repeat steps 1–4 until convergence.

To choose students for the adaptive cohort, we simply selected the nodes with the highest values of *P*_2_ that were susceptible (i.e., had not yet tested positive during the semester).

### Link prediction

The second model predicted risk at the edge (link) level by utilizing correlations between edge types to predict unobserved contact tracing relationships. Our approach to this problem, known as multi-relational link prediction, is adapted from the work of Yang et al.^19^. For each pair of nodes *u*, *v* ∈ *V* we computed the probability of a contact tracing relationship *P*(*u*, *v*) according to:

2 $$P(u,v)={C}_{\rm{katz}}(u)\left({f}_{1}(u,v)+{f}_{2}(u,v)\right),$$

where *C*_katz_(*u*) is the Katz centrality^20^ of node *u*, and *f*_1_ and *f*_2_ represent the one-hop and two-hop information flow, respectively, from *u* to *v*. We define *f*_1_ as follows:

3 $${f}_{1}(u,v)=\mathop{\sum}\limits_{t\in T}\frac{\sigma (t)}{| {{{{\mathcal{N}}}}}_{t}(u)| }\mathop{\sum}\limits_{v\in {{{{\mathcal{N}}}}}_{t}(u)}w{\left(u,v,t\right)}^{2},$$

where *σ*(*t*) is a learned conditional probability that two nodes *u* and *v* will be contact traced given that they are connected by an edge of type *t*. We compute *f*_2_ in the same manner as *f*_1_, but with respect to a two-hop neighbor graph of *G*. The two-hop neighbor graph is constructed by adding an edge of type *t* between any two nodes *u* and *v* if they are both neighbors to a common node *x* via edge type *t* (i.e., (*u*, *x*, *t*) ∈ *E* and (*x*, *v*, *t*) ∈ *E*. To learn the conditional probabilities for *σ*, we utilized a training network built from student and contact tracing data from the (previous) fall 2020 semester. For each edge of type *t*, we simply computed the probability that the pair of students connected by that edge were also identified as close contacts. This approach assumes that although student information (dorm address, course schedule, etc.) may change between semesters, the conditional probability distribution of contact tracing relationships does not.

To choose students for the adaptive cohort, we first computed *P*(*u*, *v*) for each node *u* that had tested positive within the previous four days with respect to each other node *v* ≠ *u*. Then we chose the nodes that were most likely to be contact traced to a positive node *u* that were also susceptible (i.e., had not yet tested positive during the semester).

### Implementation details

The node risk model was implemented using version 4.2 of Neo4j’s graph database. The link prediction model was implemented in Python 3.7.3 and NetworkX 2.5. All analysis was conducted using Python 3.7.3 and Pandas 1.2.

### Ethics

The University of Notre Dame Institutional Review Board (IRB) reviewed the research protocol and determined it to be exempt from human subjects research regulations (approval number: 20-12-6364). All analysis was conducted on a secure remote server in order to maintain student privacy and confidentiality.

### Reporting summary

Further information on research design is available in the Nature Research Reporting Summary linked to this article.

## Supplementary information

Reporting Summary Checklist

## Data Availability

The data used in this study are subject to privacy restrictions, but may be anonymized and made available upon reasonable request to the corresponding author.

## Code availability

The code used in this study may be made available upon reasonable request to the corresponding author.
