# Supplementary material for: Data-driven testing program improves detection of COVID-19 cases and reduces community transmission
Source: NPJ Digit Med. 2022 Feb 11;5:17. doi: 10.1038/s41746-022-00562-4 (PMC8837751; doi:10.1038/s41746-022-00562-4)
Supplement: Supplementary file 1 — Reporting Summary Checklist [file 41746_2022_562_MOESM1_ESM.pdf]

nature portfolio

Corresponding author(s): Dr Nitesh (V.) Chawla

Last updated by author(s): 2022- 01- 05

Reporting Summary

Nature Portfolio wishes to improve the reproducibility of the work that we publish. This form provides structure for consistency and transparency in reporting. For further information on Nature Portfolio policies, see our Editorial Policies and the Editorial Policy Checklist.

Statistics

For all statistical analyses, confirm that the following items are present in the figure legend, table legend, main text, or Methods section.

n/a	Confirmed	
☐	☒	The exact sample size (n) for each experimental group/condition, given as a discrete number and unit of measurement
☐	☒	A statement on whether measurements were taken from distinct samples or whether the same sample was measured repeatedly
☒	☐	The statistical test(s) used AND whether they are one- or two-sided<br>*Only common tests should be described solely by name; describe more complex techniques in the Methods section.*
☒	☐	A description of all covariates tested
☒	☐	A description of any assumptions or corrections, such as tests of normality and adjustment for multiple comparisons
☐	☒	A full description of the statistical parameters including central tendency (e.g. means) or other basic estimates (e.g. regression coefficient) AND variation (e.g. standard deviation) or associated estimates of uncertainty (e.g. confidence intervals)
☒	☐	For null hypothesis testing, the test statistic (e.g. F, t, r) with confidence intervals, effect sizes, degrees of freedom and P value noted<br>*Give P values as exact values whenever suitable.*
☒	☐	For Bayesian analysis, information on the choice of priors and Markov chain Monte Carlo settings
☒	☐	For hierarchical and complex designs, identification of the appropriate level for tests and full reporting of outcomes
☒	☐	Estimates of effect sizes (e.g. Cohen's d, Pearson's r), indicating how they were calculated

Our web collection on statistics for biologists contains articles on many of the points above.

Software and code

Policy information about availability of computer code

Data collection	The node risk model was implemented using version 4.2 of Neo4j's graph database. The link prediction model was implemented in Python 3.7.3 and NetworkX 2.5.
Data analysis	All analysis was conducted using Python 3.7.3 and Pandas 1.2.

For manuscripts utilizing custom algorithms or software that are central to the research but not yet described in published literature, software must be made available to editors and reviewers. We strongly encourage code deposition in a community repository (e.g. GitHub). See the Nature Portfolio guidelines for submitting code & software for further information.

Data

Policy information about availability of data

All manuscripts must include a data availability statement. This statement should provide the following information, where applicable:

- Accession codes, unique identifiers, or web links for publicly available datasets
- A description of any restrictions on data availability
- For clinical datasets or third party data, please ensure that the statement adheres to our policy

The data and code used in this study is subject to privacy restrictions, but may be anonymized and made available upon reasonable request to the corresponding author.

Field-specific reporting

Please select the one below that is the best fit for your research. If you are not sure, read the appropriate sections before making your selection.

☒ Life sciences ☐ Behavioural & social sciences ☐ Ecological, evolutionary & environmental sciences

For a reference copy of the document with all sections, see nature.com/documents/nr-reporting-summary-flat.pdf

Life sciences study design

All studies must disclose on these points even when the disclosure is negative.

Sample size	The sample size was not fixed but determined by which students were predicted to be high-risk by the machine learning models.
Data exclusions	No data was excluded from this study.
Replication	The COVID-19 test results and community transmission cannot be replicated. All analysis was performed independently and verified by multiple coauthors.
Randomization	Students were assigned to experimental groups based on the predictions of the machine learning models.
Blinding	Blinding was not necessary and not part of the operational requirements of the program. Students were informed of whether their COVID-19 testing appointment was for general surveillance or as part of the adaptive program.

Reporting for specific materials, systems and methods

We require information from authors about some types of materials, experimental systems and methods used in many studies. Here, indicate whether each material, system or method listed is relevant to your study. If you are not sure if a list item applies to your research, read the appropriate section before selecting a response.

Materials & experimental systems

n/a	Involved in the study	
☒	☐	Antibodies
☒	☐	Eukaryotic cell lines
☒	☐	Palaeontology and archaeology
☒	☐	Animals and other organisms
☐	☒	Human research participants
☒	☐	Clinical data
☒	☐	Dual use research of concern

Methods

n/a	Involved in the study	
☒	☐	ChIP-seq
☒	☐	Flow cytometry
☒	☐	MRI-based neuroimaging

Human research participants

Policy information about studies involving human research participants

Population characteristics	Subjects were all university students, a majority of whom were white and aged 19-23. Specific details are available in the manuscript.
Recruitment	Subjects were opted in as part of their enrollment as students at the university during the spring 2021 semester.
Ethics oversight	University of Notre Dame Institutional Review Board

Note that full information on the approval of the study protocol must also be provided in the manuscript.
